# LINKING SUPPORT PROVISION AND CHANGE IN DEPRESSIVE SYMPTOMS AMONG OLDER ADULTS OVER EIGHT YEARS

**DOI:** 10.1093/geroni/igae098.1531

**Published:** 2024-12-31

**Authors:** Debarati Kole, Heather Fuller

**Affiliations:** North Dakota State University, Fargo, North Dakota, United States; North Dakota State University, Fargo, North Dakota, United States

## Abstract

Research indicates giving support to others positively affects older adults’ overall well-being; however, limited research examines longitudinal shifts in associations between older adults’ support provision and psychological well-being over time. This study explores links between support provision and changes in depressive symptomatology in older adults over eight years and the moderating role of physical health. Data comes from the Social Integration and Aging Study, a longitudinal, community-based survey of older adults (60+) in the Upper Midwest. Data collection was conducted from 2015 (Wave 2; N= 314) to 2023 (Wave 6; N=98). Support provision was assessed with a 2-item measure of frequency of providing help to family and friends, and depressive symptoms were assessed with the 10-item CES-D scale (Cronbach’s Alpha =.82). Longitudinal Multi-Level Modelling was used to assess the time-varying effect of support provision on change in older adults’ depressive symptoms across 5 timepoints (controlling for age, gender, marital status, and subjective health). Statistically significant results were found both between-person (B=-.502 p<.001) and within-person (B=-.263 P=.022) with higher support provision predicting lower depressive symptoms; however, the interaction of time by within-person help provision (B=.133, P=.017) indicated support provision has long-term detrimental effects for depressive symptoms. Further, subjective health significantly moderated (B=.156, p<.031) the between-person association between support provision and depressive symptoms over time indicating increased progression of depressive symptoms for those with poor health/low support provision and good health/high support provision. Overall, findings indicate complex longitudinal relationships between help provision and older adults’ psychological well-being.

